# Associations of TERC Single Nucleotide Polymorphisms with Human Leukocyte Telomere Length and the Risk of Type 2 Diabetes Mellitus

**DOI:** 10.1371/journal.pone.0145721

**Published:** 2015-12-31

**Authors:** Rasha Al Khaldi, Olusegun Mojiminiyi, Fahd AlMulla, Nabila Abdella

**Affiliations:** 1 Department of Pathology, Faculty of Medicine, Kuwait University, Kuwait, Kuwait; 2 Deartment of Medicine, Faculty of Medicine, Kuwait University, Kuwait, Kuwait; University of Newcastle, UNITED KINGDOM

## Abstract

Previous Studies have mapped putative loci that may probably regulate leukocyte telomere length (LTL). The strongest associations with LTL were reported for SNP rs12696304 and rs16847897 near the non-coding Ribose Nucleic Acid (RNA) molecule component (*TERC*) of telomerase enzyme on 3q26. It is unclear whether these identified loci coding functional components of telomerase, exert a similar effect on LTL in other populations or influence risk factors of Type 2 Diabetes Mellitus (T2DM). The present study was performed to: study the influence of *TERC* polymorphisms on LTL, human telomerase reverse transcriptase (hTERT), indices of obesity and explore the potential associations with T2DM. 225 T2DM patients and 245 age and sex matched controls were studied. Allelic Discrimination (AD) genotyping was utilized to determine *TERC* SNPs [rs12696304 and rs16847897]. hTERT, adiponectin, Insulin, Homeostasis Model Assessment (HOMA-IR), and LTL were measured. Body Mass Index (BMI) and waist circumference (WC) were recorded. [CC] genotype of rs16847897 was significantly associated with shorter LTL [OR = 1.6, p = 0.004], lower hTERT levels [OR = 0.4, p = 0.006], higher BMI [OR = 2.2, p = 0.006], larger WC [OR = 23.4, p = 0.007] and hypo-adiponectemia [OR = 0.6, p = 0.006]. [GG] genotype of rs12696304 was also significantly associated with shorter LTL [OR = 1.5, p = 0.004], lower hTERT [OR = 0.7, p = 0.006] but with larger WC[OR = 5.3, p = 0.004]. [CC] genotype of rs16847897 and [GG] genotype of rs12696304 together increased the risk of T2DM significantly [OR = 1.7, p = 0.004]. We provide insights connecting a structure that is critically involved in maintaining genomic stability with obesity and T2DM. Given the central role of telomere length in determining telomere function our findings may expand our understanding of the pathological mechanisms underlying age associated conditions such as T2DM.

## Introduction

Telomeres are tandem TTAGGG repeats that cap the ends of our chromosomes preventing chromosomal fusions and genomic instability [1, 2]. Telomeres are bound by a specialized protein complex known as Shelterin and replenished by telomerase [3, 4]. Telomerase is composed of the main catalytic subunit with reverse transcriptase activity [TERT] subunit and an ubiquitously expressed non-coding Ribose Nucleic Acid (RNA) molecule complementary to the hexametric telomeric repetitions (*TERC*) subunit [5]. Telomeres and telomerase have recently been linked to several age associated diseases namely obesity and Type 2 Diabetes Mellitus (T2DM). At present, telomere shortening is widely accepted as a biomarker for age and stress related conditions [6].

Over the past decade, quantitative trait locus studies have mapped putative loci that may be involved in regulating Leukocyte Telomere Length (LTL) to human chromosomes 3q26.1, 10q26.13, and 12q12.22 [7, 8]. Indeed, a number of recent genome-wide association studies (GWAS) identified common single nucleotide polymorphisms (SNPs) near *TERC* associated with LTL in European, American, and Chinese populations [7, 9, 10]. The strongest associations with LTL were reported for SNPs rs12696304 and rs16847897 near *TERC* on 3q26 [7, 9, 10]. However, it is unclear whether the locus identified in Europeans, American, and Chinese exerts a similar effect on LTL in the Arab population and specifically in the Kuwaiti population. None of the earlier studies attempted to explore the effect of such SNPs on serum human telomerase reverse transcriptase (hTERT) levels. Hence, this study aims at exploring the associations, between the two major SNPs near *TERC* [rs16847897 and rs12696304], LTL, and hTERT levels in the Kuwaiti population. Since the link between shorter LTL and obesity is well documented we propose that SNPs near *TERC* could also affect the degree of obesity, as well as obesity related adipocytokines such as adiponectin (AdipoQ). This study also aims at exploring the influence of the two commonly reported SNPs near TERC (rs16847897 and rs12696304) on anthropometric measures, AdipoQ, and its high molecular weight isoform (HMW-AdipoQ). We aim to also investigate the potential associations of these SNPs with the incidence of T2DM.

## Methods

### 2.1 Patients and controls

As part of the protocol approved by local ethics committee in accordance with the ethical standards in Helsinki declaration, we obtained fasting 12- to -14-h blood samples from 225 T2DM patients (115 females, 110 males) who were recruited from Mubarak Al-Kabeer Hospital. Diagnosis of T2DM was based on World Health Organization (WHO) criteria [11]. 245 (111 females and 134 males) age- and sex- matched healthy subjects were enrolled from the Kuwait Blood Bank Center. The study protocol as approved by the ethics committees of the Ministry of Health and the Faculty of Medicine was explained thoroughly by a consultant physician (Nabila Abdella) to the subjects who were then asked to sign informed voluntary consent form in order to participate in the study. Only subjects who gave consent to participate in the study were included in this study. Patients were given free access to the study protocol and were free to ask questions related to the use of their blood samples for the study. Subjects who needed time to consider their potential participation were given sufficient time to do so and were only recruited after obtaining their consent. As per local laws, all data were kept confidential and protected. All participants had the right to withdraw from the study unconditionally at any time without giving any reasons. The care they received while in the hospital, and afterwards were not affected in any way if the participants had chosen not to participate or withdraw from the study at a later time. Informed voluntary verbal consent to participate in the study were obtained. The ethics committees of the Ministry of Health and the Faculty of Medicine, Kuwait University approved and accepted the use of verbal informed voluntary consent obtained from study subjects. Subjects were identified as Kuwaitis by their civil identification cards.

### 2.2 Anthropometric measures

Body weight (Kg) was measured in light clothing without shoes and height (cm) was measured as the distance from the top of the head to the bottom of the feet using a fixed stadiometer. Subsequently, body mass index (BMI) [Kg/m^2^] as an index of general obesity, was calculated according to the following formula: BMI = Weight (Kg) / Height (m^2^). Waist circumference (WC) (cm) at the level of the umbilicus was also taken. Blood pressure was measured in the right arm of a seated participant by using a standard gauge mercury column sphygmomanometer after resting for at least 5 minutes. Hypertension was defined as systolic blood pressure ≥ 140 mm Hg, diastolic blood pressure ≥90 mm Hg and/or current history of antihypertensive medication [12]. All the previous measurements were taken by one investigator (Rasha M Al-Khaldi) only, to exclude any inter-observer variability.

### 2.3 Biochemical analysis

The collected blood samples were immediately chilled on ice and centrifuged at 3000 RPM for 10 minutes in a cold centrifuge. The harvested plasma, serum, and blood cells were stored frozen at -20°C for subsequent analysis in appropriate 300 μL labeled aliquots to avoid frequent freezing and thawing. Serum levels of hTERT were determined using (Genway Biotech Inc., San Diego, USA). The plasma AdipoQ concentrations were measured by Enzyme Linked Immunosorbent Assay Kit [ELISA] (Linco Research, Missouri, USA). HMW-AdipoQ was measured in plasma using AdipoQ Mulitmeric Sandwich ELISA (ALPCO^™^ Diagnostics, New Hamshire, USA). Performance characteristics of each ELISA Kit used in this study are summarized in “S1 Table”. Serum levels of insulin were measured using (Siemens Healthcare Diagnostics, Deerfield, USA). Degree of insulin resistance (IR) was assessed using the homeostatic model assessment (HOMA-IR) Calculator software version 2.2.2 for Windows [Diabetes Trials Unit, University of Oxford, at: http://www.dtu.ox.ac.uk/index.htm?maindoc=/publications/ [13]. Fasting plasma glucose (FPG), total cholesterol (TC), triglycerides (TG), and high density lipoprotein cholesterol (HDL-Chol), were measured using UniCel^®^ DxC Synchron 800 analyzer (Beckman Corporation, Brea, CA, USA). Low density lipoprotein cholesterol (LDL-Chol) was calculated using Friedewald formula. Glycated hemoglobin (HbA1c) was measured on TOSOH G8 High Performance Liquid Chromatography Analyzer (TOSOH Bioscience, California, USA) with dedicated reagents.

### 2.4 DNA extraction

Maxwell^®^ 16 Blood DNA Purification Kit (Promega was used to extract deoxyribonucleic acid (DNA) from whole blood samples. DNA quantity and quality were checked using Nano-drop spectrophotometer (Thermo Fisher Scientific Inc, Massachusetts, USA). The expected range of high quality DNA preparations based on the A260/A280 nm wavelength ratio should be ≥ 1.7. Ratios of samples which deviated from this range, indicated protein, or RNA contamination, and, re-purification of DNA was performed until satisfactory.

### 2.5 Telomere length measurement

Singleplex quantitative polymerase chain reaction [qPCR] was used to measure LTL. The method involved determining the telomere to single copy gene [T/S] ratio in comparison to reference DNA to yield relative T/S ratios proportional to average telomere length [14]. Reactions for telomere and single-copy control gene [S] [36B4, acidic ribosomal phosphoprotein PO, located on chromosome 12] were run in duplicates in 25 μl on 7500 Fast Real Time PCR (Applied Biosystems, California, USA) (Detailed protocol, “S1 Text”). All samples were analyzed in a blinded fashion without knowledge of the clinical data.

### 2.6 Genotyping of SNPs near *TERC*

Genotyping of the candidate *TERC* SNPs (rs16847897 and rs12696304) was carried out by allelic discrimination [AD] using pre-designed TaqMan^®^ SNP genotyping assays (Applied Bio-systems, California, USA). The protocol of Applied Biosystems was followed carefully. The results of automated allele calling were verified by examining the amplification plots and the Ct values of each sample.

### 2.7 Statistical analysis

Data were tested for normality using the Kolmogorov Smirnov test. Non-parametric variables were normalized by log transformation to use parametric tests, otherwise non-parametric tests were used. Descriptive statistics, namely mean±SD, 95% confidence interval (CI) or median and inter-quartile range were used as appropriate. Comparison between mean values in the 2 groups (Patients and Controls) was evaluated using students *t* test. For each SNP, Hardy-Weinberg Equilibrium was assessed using the following facility: www.oege.org/software. The differences in LTL, hTERT, anthropometric and biochemical findings between *TERC* genotypes of the two SNPs were evaluated by one-way analysis of variance (ANOVA). The association between *TERC* genotypes and variables of interest was assessed using multivariate logistic regression analysis. The association between *TERC* genotypes and T2DM risk was determined by calculating the odds ratio (OR) and 95% confidence interval (CI) using binary logistic regression. Logistic regression analyses were adjusted for any confounders, such as age and sex. linear regression were used to determine the association between gentotypes of the two SNPs, telomere length, telomerase and obesity related factors. For that purpose, Beta coefficients (β) with 95% confidence interval (CI) were presented. Haplotypes and their frequencies as well as the presence of Linkage disequilibrium were inferred using http://bioinfo.iconcologia.net/snpstats/start.htm. All the statistical analyses were performed with International Business Machines Corporation Statistical Package for Social Sciences (IBM SPSS Statistics, New York, USA), version 21. The criterion for statistical significance was p<0.05.

## Results

### 3.1 Demographic, anthropometric and biochemical characteristics of the study population

A total of 225 T2DM patients and 245 controls were included in this study. Table 1 summarizes the demographic and anthropometric characteristics of the study population according to their status (controls versus T2DM). T2DM patients had significantly higher BMI and WC than control subjects. Not surprisingly, T2DM patients had significantly higher HbA1c, FPG and TG but lower HDL-Chol compared to control subjects. T2DM patients had significantly shorter LTL and lower levels of hTERT as shown in Table 1. Frequencies of metabolic syndrome in the study population according to the three major definitions of metabolic syndrome are shown in “S2 Table”.

**Table 1:** Demographic, anthropometric and biochemical characteristics of the study population.

	Apparently Healthy Control Subjects [n = 245]	T2DM Patients [n = 225]	
Parameter	Mean±SD	Mean±SD	*p*-value
Age [Years]	52.9±9.4	54.7±9.9	NS
BMI [Kg/m^2^]	29.0±7.8	32.4±6.3	0.001
WC [cm]	101.8±15.8	111.2±12.2	<0.0001
Sys BP [mm/Hg]	124±16.0	136.0±21.0	<0.0001
Dia BP [mm/Hg]	78.0±10.0	82.0±11.0	<0.0001
HbA1c [%]	5.9±1.3	9.3±1.3	<0.0001
FPG [mmol/L]	5.2±0.96	9.8±3.9	<0.0001
TC [mmol/L]	5.0±1.2	4.7±1.0	0.002
TG [mmol/L]	1.2±1.9	1.6±1.9	0.03
HDL-Chol [mmol/L]	1.5±0.4	1.0±0.3	0.002
LDL-Chol [mmol/L]	3.1±0.9	2.9±0.8	0.001
LTL [T/S ratio]	4.2±0.24	3.5±0.19	0.01
hTERT [ng/mL]	32.9±8.85	21.3±4.74	<0.0001

BMI = Body Mass Index, WC = Waist Circumference, BP = Blood Pressure, Sys = Systolic, Dia = Diastolic, HbA1c = Glycated Hemoglobin A1c, FPG = Fasting Plasma Glucose, TC = Total Cholesterol, TG = Triglycerides, HDL-Chol = High Density Liporpotein Cholesterol, LDL-C = Low Density Lipoprotein Cholesterol, LTL = Leukocyte Telomere Length, hTERT = Human Telomerase Reverse Transcriptase.

### 3.2 Prevalence rates of *TERC* genotypes

SNPs rs16847897 and rs12696304 were genotyped in all the 470 samples. Neither of the two SNPs showed a statistical deviation from Hardy—Weinberg equilibrium [*p* value = 0.22 and 0.32] respectively. The [*C*] allele of rs16847897 and [*G*] allele of rs12696304 frequencies were 35.6% and 35.9% respectively in this study as shown in “S3 Table”.

### 3.3 Anthropometric and biochemical characteristics according to *TERC* genotypes

Homozygous carriers of minor allele *C* [*CC*] of rs16847897 had significantly larger WC compared to [*GC*] or [*GG*] genotypes as shown in Table 2. On the other hand, [*GG*] genotype of rs12696304 tended to have slightly larger WC compared to [*CC*] or [*GC*] genotypes. Carriers of minor allele *C* of rs16847897 had significantly higher FPG, HbA1c% and TG levels compared to [*GC*] or [*GG*] genotypes. Homozygous carriers of [*G*] allele of rs12696304 showed similar trends.

**Table 2:** Anthropometric and biochemical characteristics according to *TERC* SNPs rs16847897 and rs12696304 genotypes in the study population.

	rs16847897				rs12696304			
	CC	GC	GG		CC	GC	GG	
Parameter	Mean±SD	Mean±SD	Mean±SD	*p*-value	Mean±SD	Mean±SD	Mean±SD	*p*-value
BMI [Kg/m2]	29.4±9.3	30.2±8.7	28.5±6.7	NS	31.2±0.3	30.9±0.5	30.9±0.5	NS
Waist Circumference [cm]	106.1±17.1	102.5±17.1	100.0±14.5	0.03	105.7±15.1	105.6±15.4	107.1±14.6	0.02
HbA1c%	7.5±2.5	7.3±2.4	7.4±2.3	0.03	7.5±2.6	8.1±2.7	8.2±2.7	0.003
FPG[mmol/L]	7.4±3.7	7.1±3.9	7.1±3.5	0.01	6.9±3.0	7.7±3.9	7.8±3.5	0.02
TC [mmol/L]	4.8±1.0	4.8±1.0	4.8±1.0	NS	4.1±1.0	3.8±1.0	4.8±1.0	NS
TG[mmol/L]	1.5±0.9	1.7±1.4	1.5±0.9	0.01	1.5±0.8	1.7±1.4	1.5±0.9	NS
HDL-Chol [mmol/L]	1.1±0.4	1.1±0.4	1.1±0.4	NS	1.1±0.4	0.9±0.4	1.1±0.4	NS
LDL-Chol [mmol/L]	3.1±0.8	3.0±0.9	2.9±0.8	NS	3.1±0.8	3.1±0.8	3.1±0.8	NS
LTL [T/S ratio]	1.1±0.8	1.8±3.2	2.04±6.40	0.04	3.2±0.1	1.8±3.2	0.8±0.1	0.02
hTERT [ng/mL]	22.7±5.9	28.8±7.9	30.1±7.6	<0.0001	28.0±1.2	24.7±1.4	14.6±2.3	<0.0001
AdipoQ [ng/mL]	5.3±2.3	5.9±2.5	6.5±2.6	0.03	6.7±2.6	5.9±2.5	5.1±2.3	0.02
HMW-AdipoQ [ng/mL]	1.2±0.9	1.8±0.9	2.5±0.9	0.04	2.8±0.9	1.8±0.9	1.1±0.9	0.03
Insulin [μU/mL]	12.4±2.9	12.5±7.9	12.2±2.3	NS	13.1±2.7	12.5±2.6	12.4±2.5	NS
HOMA-IR	4.2±2.5	4.0±2.5	4.1±2.4	NS	3.8±2.1	4.5±2.7	4.4±2.2	NS

BMI = Body Mass Index, WC = Waist Circumference, HbA1c = Glycated Hemoglobin A1c, FPG = Fasting Plasma Glucose, TC = Total Cholesterol, TG = Triglycerides, HDL-Chol = High Density Liporpotein Cholesterol, LDL-Chol = Low Density Lipoprotein Cholesterol, LTL = Leukocyte Telomere Length, hTERT = Human Telomerase Reverse Transcriptase, AdipoQ = Adiponectin, HMW-AdipoQ = High Molecular Weight Adiponectin, HOMA-IR = Homeostasis Model Assessment Insulin Resistance.

### 3.4 Variations in LTL, hTERT levels and other obesity related parameters with TERC genotypes

[*CC*] genotype of rs16847897 had significantly the shortest LTL and the lowest hTERT levels among the 3 genotypes of rs16847897. [*GG*] genotype of rs16847897 had significantly higher AdipoQ and HMW-AdipoQ levels compared to [*GC*] or [*CC*] genotypes. Though no significant differences in insulin levels were observed between the 3 genotypes, homozygous carriers of minor allele *C* of rs16847897 had significantly higher HOMA-IR compared to [*GC*] or [*GG*]. Homozygous carriers of the minor allele *G* [*GG*] of SNP rs12696304 had significantly the shortest LTL and the lowest hTERT levels compared to the other two identified genotypes. On the other hand, [*CC*] genotype had significantly higher total AdipoQ. Moreover, [*GG*] genotype had significantly the highest IR presented by HOMA-IR as shown in Table 2 compared to other genotypes.

### 3.5 *TERC* genotypes and Logistic regression analysis

Using binary logistic regression analysis, it was found that homozygous carriers of the C allele of rs16847897 were at risk of the 1.6 folds of having shorter LTL compared to those carrying G alleles [*GG*]. Additionally, [*CC*] genotype was significantly associated with lower levels of hTERT as shown in Table 3. We also found that [*CC*] genotype was significantly associated with higher BMI, WC and lower levels of total AdipoQ and HMW-AdipoQ levels. [*GG*] genotype of rs12696304 was significantly associated with increased risk of shorter LTL and lower levels of hTERT as shown in Table 3. Furthermore, [*GG*] genotype of SNP rs12696304 was significantly associated with higher BMI, WC, lower total AdipoQ and HMW-AdipoQ levels. In the parallel negative association between [*GG*] genotype of rs12696304 and AdipoQ, we found that [*GG*] genotype of rs12696304 was significantly associated with higher HOMA-IR as shown in Table 3. Beta Coefficients [95CI] for associations of TERC SNPs rs16847897 and rs12696304 with LTL, hTERT, anthropometric indices and metabolic factors of obesity as shown in “S5 Table”.

**Table 3:** ORs [95CI] for associations of *TERC* SNPs rs16847897 and rs12696304 with LTL, hTERT, anthropometric indices and metabolic factors of obesity.

	LTL	hTERT	BMI	WC	AdipoQ	HMW-AdipoQ	Insulin	HOMA-IR
**rs16847897**								
GG	1	1	1	1	1	1	1	1
GC+CC	1.4**[1.3–1.5]	0.7**[0.6–0.8]	1.6[0.9–2.7]	7.4**[4.1–13.3]	0.8*[0.7–0.9]	0.5**[0.5–0.7]	1.0[0.9–1.1]	2.0*[1.8–2.1]
GC+GG	1	1	1	1	1	1	1	1
CC	1.6**[1.5–1.7]	0.4**[0.4-.06]	2.2**[1.5–3.1]	23.4**[15.6–35.2]	0.6**[0.5–0.9]	0.6**[0.5–0.7]	1.2**[1.2–1.4]	2.4*[2.1–2.7]
**rs12696304**								
CC	1	1	1	1	1	1	1	1
GC+GG	1.3**[1.3–1.6]	0.7** [0.6–0.8]	1.0[0.9–1.1]	0.5[0.3–1.1]	0.7**[0.6–0.8]	0.6**[0.5–0.7]	1.1*[1.1–1.3]	1.3*[1.2–1.4]
GC+CC	1	1	1	1	1	1	1	1
GG	1.5**[1.4–1.6]	0.4**[0.3–0.5]	1.2**[1.1–1.3]	5.3**[3.5–8.0]	0.5**[0.5–0.7]	0.7**[0.5–0.7]	1.5**[1.3–1.6]	1.2*[1.1–1.3]

* *p*<0.05,

** *p*<0.01,

*** *p*<0.001,

**** *p*<0.0001,

No * = No Significance.

LTL = Leukocyte Telomere Length, hTERT = Human Telomerase Reverse Transcriptase, BMI = Body Mass Index, WC = Waist Circumference, AdipoQ = Adiponectin, HMW-AdipoQ = High Molecular Weight Adiponectin, HOMA-IR = Homeostasis Model Assessment Insulin Resistance.

### 3.6 Haplotype-analysis

**Fig 1:**
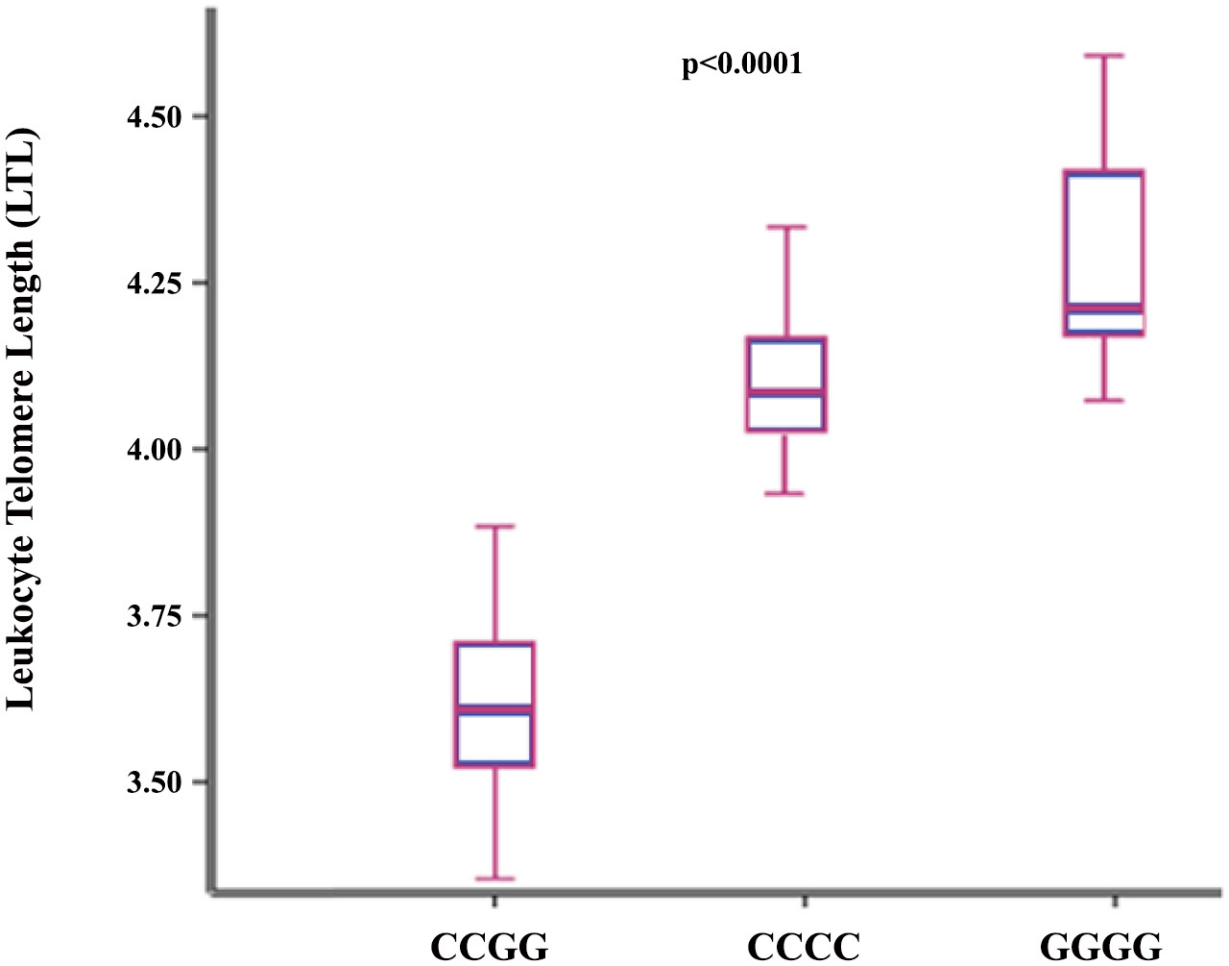
Box plots show the distribution of Leukocyte Telomere Length [LTL]. The horizontal line within the box corresponds to the median value. Vertical bars represent the values between 2.5^th^ to 97.5^th^ percentiles excluding outliers.

**Fig 2:**
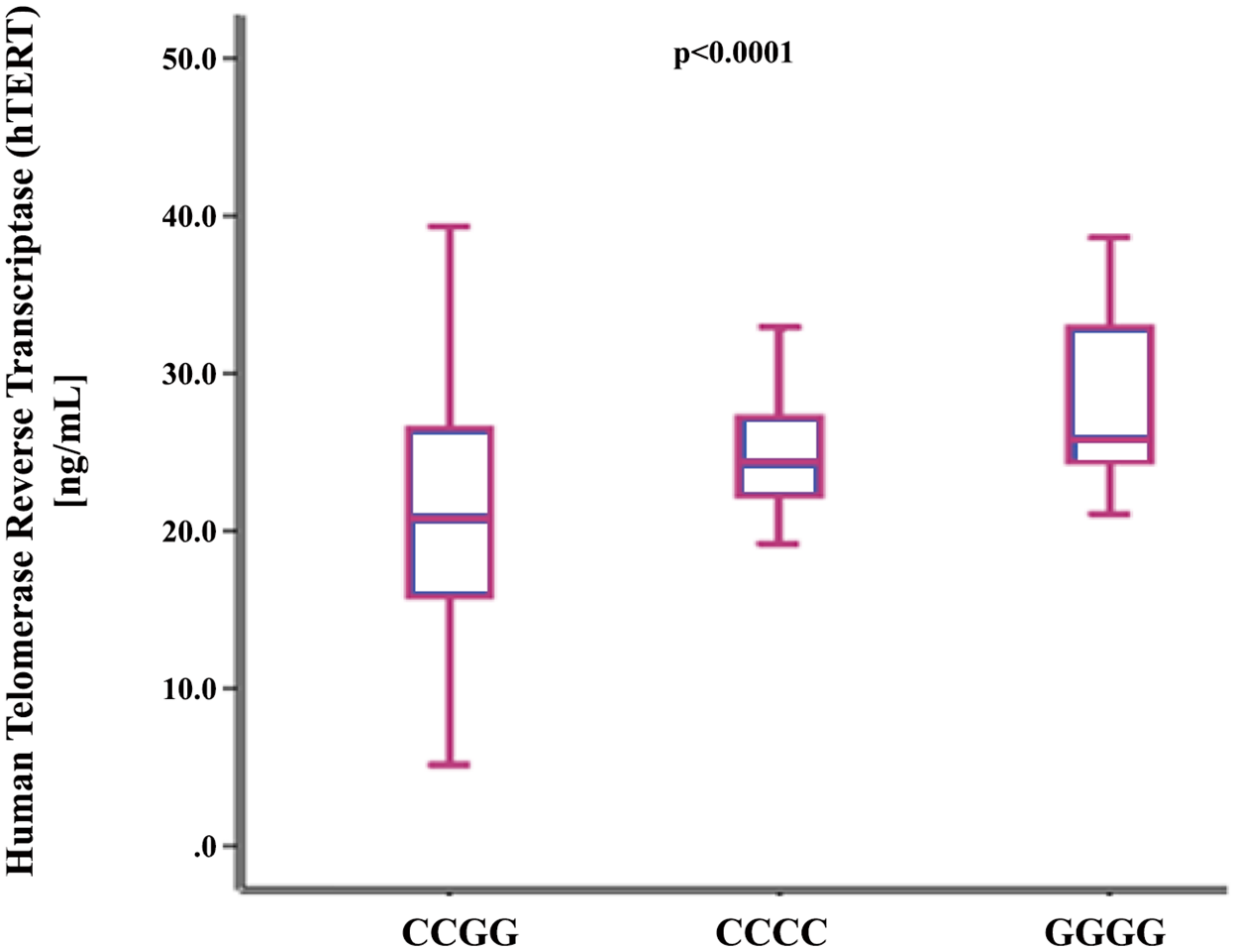
Box plots show the distribution of serum levels of human telomerase reverse transcriptase [hTERT]. The horizontal line within the box corresponds to the median value. Vertical bars represent the values between 2.5^th^ to 97.5^th^ percentiles excluding outliers.

The two *TERC* SNPs were in high, although not in perfect, linkage disequilibrium (LD) in the study population [D’ = 0.839, r^2^ = 0.687]. [*CG*] haplotype had significantly higher BMI and WC compared to [*CC*] and [*GG*] haplotypes “S6 Table”. LTL and hTERT levels varied significantly among the 3 identified haplotypes (S7 Table). Indeed, LTL was the shortest and hTERT levels were the lowest in [*CG*] haplotypes as shown in Figs 1 and 2. We found that [*CG*] haplotype was significantly associated with higher risk of shorter LTL and lower telomerase levels (Table 4). On the other hand, [*CC*] haplotype was significantly associated with higher hTERT levels but not with LTL. Additionally, [*GG*] haplotype was found to be associated with lower risk of shorter LTL and higher levels of hTERT in contrast to [*CG*] haplotype as shown in Table 4. [*CG*] haplotype was significantly associated with larger WC. In accordance with the high WC, [*CG*] haplotype was significantly associated with higher HOMA-IR. [*CC*] and [*GG*] haplotypes showed the opposite trends to haplotype [*CG*]. In fact [*CC*] haplotype was significantly associated with higher total AdipoQ and HMW-AdipoQ levels (Table 4). This probably explains the negative association reported in those haplotypes with HOMA-IR and insulin levels.

**Table 4:** ORs [95%CI] for associations of haplotypes with LTL, hTERT, anthropometric indices and metabolic factors of obesity.

	LTL	hTERT	BMI	WC	AdipoQ	HMW-AdipoQ	Insulin	HOMA-IR
GCGC	1	1	1	1	1	1	1	1
CCGG	1.6**[1.4–1.7]	0.4*[0.2–0.80]	0.9*[0.8–0.9]	7.1**[3.8–13.1]	1.1*[1.1–1.2]	0.6**[0.5–0.7]	1.2**[1.2–1.3]	2.6*[2.2–3.0]
CCCC	0.87[0.7–1.1]	2.3**[1.9–2.8]	0.5**[0.4–0.6]	0.8[0.7–0.9]	21.1**[2.3–22.0]	79.7**[42.7–50.0]	0.4**[0.3–0.6]	0.5**[0.49–0.59]
GGGG	0.4**[0.49–0.59]	0.7**[0.7–0.9]	1.9**[1.2–3.1]	6.8**[5.3–8.9]	0.7**[0.7–0.9]	0.8**[0.7–0.9]	2.7**[2.1–3.6]	0.4**[0.3–0.6]

* *p*<0.05,

** *p*<0.01,

*** *p*<0.001,

**** *p*<0.0001,

No * = No Significance.

BMI = Body Mass Index, WC = Waist Circumference, LTL = Leukocyte Telomere Length, hTERT = Human Telomerase Reverse Transcriptase, AdipoQ = Adiponectin, HMW-AdipoQ = High Molecular Weight Adiponectin, HOMA-IR = Homeostasis Model Assessment Insulin Resistance.

### 3.7 Association of SNPs and haplotypes with T2DM risk

We found that [*CC*] genotype rs16847897 and [*GG*] genotype of rs12696304 were significantly associated with higher risk of T2DM [OR = 1.6[1.5–1.9], *p* = 0.005]. On the other hand, we found that [*CG*] haplotype was associated significantly with higher risk of T2DM [OR = 1.5[1.3–1.9], *p* = 0.007]. On the contrary, [*CC*] and [*GG*] haplotypes were associated with lower risk of T2DM [OR = 0.2[0.1–0.6], *p* = 0.03, OR = 0.3[0.1–0.6], *p* = 0.02] respectively.

## Discussion

Homozygous carries of risk alleles [*C*] of rs16847897 and [*G*] of rs12696304 were found to have shorter LTL compared with other genotypes in our study. Our results are in agreement with what has been found in European, American and Chinese Han populations [7, 9, 10]. The frequencies of shorter LTL risk alleles [*C*] and [*G*] were similar to those reported in Europeans and Americans, but contrasted to those found in the Chinese Han population [9]. Indeed, the risk alleles of rs16847897 [*C*] and rs12696304 [*G*] were major alleles in the Chinese Han population. These discrepancies can be explained by ethnic/racial differences. Genetic drift that occurs in all populations may also explain the differences in allele frequencies reported in different populations. These findings are important in understanding the effect of evolution on population genetics and designing replication studies to identify risk alleles of short LTL in different ethnic groups. In the future, collaborative efforts of researchers with different ethnicities will identify genetic variants that are relatively common across populations and unravel the interactions among these susceptibility variants and also with environmental exposures that modulate LTL. Rather than considering each marker individually, specific combinations of allelic variants in a series of tightly linked markers on the same chromosome, i.e. haplotypes, were tested. To the best of our knowledge, our study is the first to examine the association of haplotypes, LTL, hTERT levels, anthropometric indices, and metabolic factors of obesity. In this study, we showed a strong LD between rs16847897 and rs12696304 [D’ = 0.84, r^2^ = 0.69] in contrast to the weak LD found in the Chinese Han population [D’ = 0.62, r^2^ = 0.26] “S1 Fig”. The strong LD found in our study simply refers to a strong correlation of tightly linked variants and hence lead to cost savings for future association studies in the field of telomere biology. Risk alleles of both SNPs rs16847897 and rs12696304 were not only associated with higher risk of shorter LTL but additionally, were significantly associated with lower levels of hTERT, an active subunit of telomerase and a major regulator of LTL. In fact, the negative association between risk alleles of SNPs rs16847897, rs12696304 and hTERT levels strongly suggests that those genetic factors may play a role in regulating levels of telomerase. In this regard, the negative association found in our study between [*CG*] haplotype and lower hTERT levels is not surprising since this haplotype is composed of the major risk alleles associated with lower hTERT levels. Therefore, we strongly suggest that the reported SNPs may actually affect telomerase levels rather than affecting LTL directly. Our assumption is supported by the telomere hypothesis, which states that in cells without telomerase activity, loss of telomere DNA will eventually trigger a cellular response, via DNA damage response, that leads to growth arrest or apoptosis. The essential role of telomerase in maintaining LTL is also supported by the observation that ectopically introduced telomerase activity can extend telomeres and indefinitely prolong cellular lifespan. More direct support for the role of hTERT in maintaining cell survival was afforded by the introduction of hTERT into primary human cells that already contained the telomerase RNA, which resulted in restoring the proliferating capacity of such cells through elongating telomeres [15]. Though the exact mechanisms by which those SNPs (rs16847897 and rs12696304) are linked to hTERT levels are still vague, they may alter transcription, post-transcriptional or translational activity or induce changes in the tertiary structure of the gene product human telomerase [7, 16]. In that essence, the effect of such SNPs can be mediated through one of the other genes in 3q26 locus that were found to actually play a role in controlling expression either by activating or repressing the transcription of various genes such as encoding myoneurin (*MYNN*) including telomerase. Region 3q26 was also found to harbor genes encoding for cell surface proteins such as three members of the Leucin-rich repeat containing (*LRCC*) super-family (*LRRC34*, *LRRC31* and *LRRIQ4*) that are essential to mediate protein to protein interactions essential for mediating the downstream effects of telomerase on LTL [7, 16], [17, 18]. The negative association among risk alleles of SNPs rs16847897 and rs12696304, LTL and hTERT levels may also be attributed to the fact that homozygous carriers of C allele and G allele respectively had higher indices of obesity namely, BMI and WC. Our finding is further supported by a recent study that has shown a negative association between anthropometric measures of obesity and LTL [19]. Evidence for the presence of linkage of 3q26 with BMI was reported in many different populations. Several genes were found to be located in this region such as: Trans-membrane 4 super-family member 1 (*TM4SF1*), and Ring-finger protein 13 [*RNF13*] which can be in LD with the studied SNPs. The effects of near *TERC* gene polymorphisms on obesity related metabolic factors have also been studied. Homozygous carriers of risk alleles of both SNPs that were associated with shorter LTL and lower hTERT levels mentioned earlier had the lowest AdipoQ, [also mapped to loci 3q26] levels. The presence of hypo-adiponectemia in [*CC*] genotype of rs16847897 and [*GG*] genotype of rs12696304 may be explained by the high WC in individuals carrying those genotypes. Our assumptions are in agreement with a previous study in the Kuwaiti population, Mojiminiyi et al., 2007, reported a negative correlation between anthropometric indices of obesity and AdipoQ [20]. Another possible explanation of the negative association among risk alleles of SNPs rs16847897 and rs12696304, AdipoQ and HMW-AdipoQ is the high index of HOMA-IR, in subjects carrying those genotypes. Studies on Pima Indians have shown that high adiponectin levels are protective against the impairment of glucose metabolism reducing the risk of developing T2DM [21, 22]. Mojiminiyi and colleagues (2007) reported also that adiponectin levels in T2DM patients were closely related to IR and the components of the metabolic syndrome [20]. Unfortunately, most if not all of the published reports examined the potential involvement of telomere-associated pathway gene variation with various types of cancer and to the best of our knowledge none of the risk of T2DM. This lack of information on the relevance of telomere pathway genes with T2DM not only makes a cross- reference comparison with the present findings difficult, but signifies the need of further investigations into the importance of telomere-pathway genes in age related disorders such as T2DM. Nevertheless, the effects of the studied SNPs on different biochemical analytes discussed previously may account partially for the association of certain genotypes of rs16847897 and rs12696304 with the risk of T2DM in this study. In this regard, [*CC*] of rs16847897 and [*GG*] of rs12696304 genotypes were significantly associated with IR, which lies at the core of the pathogenesis of T2DM. Hence, not surprisingly, in this study, the odds ratio of [*CG*] and [*CC*] of rs16847897 confirmed that both of these genotypes have an elevated risk of T2DM. Moreover, the negative association of the risk alleles *C* and *G* of both SNPs rs16847897 and rs12696304 with AdipoQ, an anti diabetic adipocytokine can also explain the higher risk of individuals carrying two copies of risk alleles for T2DM. Not surprisingly, the association between [*CG*] haplotype and the higher risk of T2DM may be attributed also to the negative association of this haplotype with AdipoQ levels. Subjects carrying [*CC*] and [*GG*] haplotypes in our study had a lower risk of T2DM probably due to the positive association of earlier mentioned haplotypes and higher levels of protective total AdipoQ and HMW-AdipoQ. The effects of SNPs may also be based on direct genetic effects, or gene-environment interactions. In fact epistasis [gene-gene] interaction has been shown to play an important role in human genetics [23]. In support of this, region 3q26 was found to harbor candidate genes that were associated significantly with the pathogenesis of T2DM such glucose transporter 2 (*GLUT2*), Apolipoprotein D (*APOD*), and Adiponectin [24]. However, before any conclusions can be made regarding the effects of genetic polymorphisms on T2DM, a clear statistical picture of the normal genotypes’ frequencies should be established for each ethnic and regional population group under study. Studies exploring the possible differences in the functional implications of different genotypes and haplotypes on the gene product are also needed.

## Conclusions

**Fig 3:**
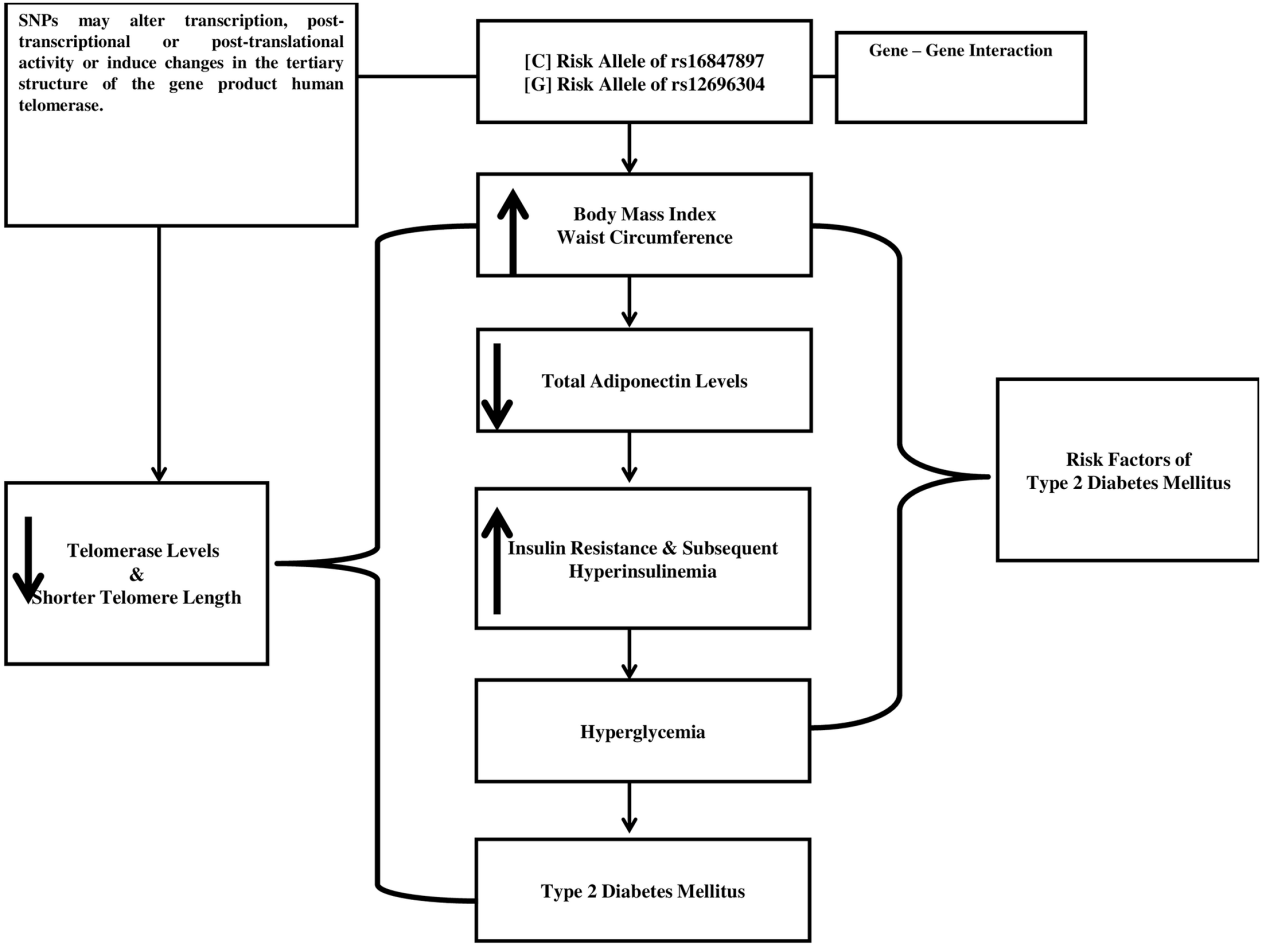
Complex interplay between TERC SNPs, short telomeres, telomerase and obesity related factors.

We have provided insights into the genetic determination of a structure that is critically involved in maintaining genomic stability. Given the importance of telomeres in cellular function and the central role of telomerase in determining telomere function, our findings may have broad relevance for both physiological and pathological age associated mechanisms. To date our study is the first in Kuwait and probably in the world that investigated the association of common *TERC* SNPs, LTL, hTERT levels, anthropometric measures, and AdipoQ. Our data show the complex interplay between TERC SNPs, short telomeres, telomerase and obesity related factors as shown in Fig 3. Presumably SNPs in telomere pathway genes may alter their function or regulation, in a manner that accelerates the telomere loss phenotype in certain complex disorders such as T2DM. Our results also strongly suggest that non-coding regions such as *TERC* genes can harbor genetic variants of clinical relevance. The identified haplotypes in this research probably captured much of the correlation between the two studied SNPs holding the key to better understanding of the role of genetic variants in the pathogenesis of complex diseases such as T2DM.

## Supporting Information

S1 Fig SNP annotation map showing the locations of both studied SNPs (rs16847897 and rs12696304). (TIF)

S1 Table Performance characteristics of ELISA kits used to measure analytes of interest in this study. (DOCX)

S2 Table Frequency of metabolic syndrome in the study population. (DOCX)

S3 Table The distribution of *TERC* genotypes within the study population. (DOCX)

S4 Table Frequency of metabolic syndrome in the study population according to *TERC* genotypes. (DOCX)

S5 Table Beta Coefficients [95CI] for associations of *TERC* SNPs rs16847897 and rs12696304 with LTL, hTERT, anthropometric indices and metabolic factors of obesity. (DOCX)

S6 Table Anthropometric and biochemical characteristics according to haplotypes. (DOCX)

S1 Text Detailed protocols for Leukocyte Telomere Length (LTL) measurement and genotyping SNPs near *TERC* using Allelic Discrimination. (DOC)

## Abbreviations

AD: Allelic Discrimination
AdipoQ: Adiponectin
ANOVA: One-way analysis of variance
APOD: Apolipoprotein D
β: Beta Coeffecients
BMI: Body Mass Index
CI: Confidence Interval
Ct: Cycle Threshold
CV: Coefficient of Variation
DNA: Deoxyribonucleic acid
ELISA: Enzyme Linked Immunosorbent Assay
FPG: Fasting Plasma Glucose
GLUT2: Glucose Transporter 2
GWAS: Genome Wide Association Studies
HbA1c: Glycated Hemoglobin
HDL-Chol: High Density Lipoprotein Cholesterol
HMW-AdipoQ: High Molecular Weight Adiponectin
HOMA-IR: Homeostasis Model Assessment—Insulin Resistance
hTERT: Human Telomerase Reverse Transcriptase
IR: Insulin Resistance
LD: Linkage Disequilibrium
LDL-Chol: Low Density Lipoprotein Cholesterol
LTL: Leukocyte Telomere Length
MPW: Millipore Water
MYNN: Encoding Myoneurin
OR: Odds Ratio
qPCR: Quantataive Real Time Polymerase Chain Reaction
RNA: Ribose nucleic acid
RNF13: Ring Finger Protein 3
S: Single Copy Gene
SNPs: Single Nucleotide Polymorphisms
SPSS: Statistical Package for Social Sciences
T: Telomere
T2DM: Type 2 Diabetes Mellitus
TC: Total Cholesterol
TERC: Non-coding Ribose nucleic acid (RNA) molecule component
TERT: Telomerase Reverse Transcriptase
TG: Triglycerides
TM4SF1: Transmembrane 4 Super Family Member 1
WC: Waist Circumference
WHO: World Health Organization
