# Supplementary material for: Associations of TERC Single Nucleotide Polymorphisms with Human Leukocyte Telomere Length and the Risk of Type 2 Diabetes Mellitus
Source: PLoS One. 2015 Dec 31;10(12):e0145721. doi: 10.1371/journal.pone.0145721 (PMC4705103; doi:10.1371/journal.pone.0145721)
Supplement: S1 Table — (DOCX) [file pone.0145721.s002.docx]

| **Serum hTERT** | |
| --- | --- |
| Sensitivity | 0.69 ng/μl |
| Inter - CV at concentration = 30.5 ng/μl | 5.0% |
| Intra – CV at concentration = 30.5 ng/μl | 5.1% |
| **Plasma total AdipoQ** | |
| Sensitivity | 2.0 ng/ml |
| Inter - CV at concentration = 15.7 ng/ml | 4.0% |
| Intra – CV at concentration = 15.7 ng/ml | 4.1% |
| **Serum Insulin** | |
| Sensitivity | 2.0 μIU/ml |
| Inter - CV at concentration = 12.7 μIU/ml | 6.7% |
| Intra – CV at concentration = 12.7 μIU/ml | 5.7% |

**S2 Table: Performance characteristics of ELISA kits used to measure analytes of interest in this study.**

hTERT= Human Telomerase Reverse Transcriptase, CV= Coefficient of Variation,

AdipoQ= Adiponectin
