# Supplementary material for: Associations of TERC Single Nucleotide Polymorphisms with Human Leukocyte Telomere Length and the Risk of Type 2 Diabetes Mellitus
Source: PLoS One. 2015 Dec 31;10(12):e0145721. doi: 10.1371/journal.pone.0145721 (PMC4705103; doi:10.1371/journal.pone.0145721)
Supplement: S2 Table — (DOCX) [file pone.0145721.s003.docx]

**S3 Table: Frequency of metabolic syndrome in the study population.**

|  | **Apparently Healthy Control**  **Subjects (n=245)** | **T2DM Patients**  **(n=225)** |
| --- | --- | --- |
| **WHO** | | |
| Metabolic Syndrome Positive | 98 (39.5%) | 195 (85.8%) |
| Metabolic Syndrome Negative | 147 (60.0%) | 30 (13.3%) |
| **ATPIII** | | |
| Metabolic Syndrome Positive | 113 (46.1%) | 202 (88.9%) |
| Metabolic Syndrome Negative | 132 (53.9%) | 23 (10.2%) |
| **IDF** | | |
| Metabolic Syndrome Positive | 124 (50.6%) | 208 (91.1%) |
| Metabolic Syndrome Negative | 121 (49.4%) | 17 (7.6%) |

WHO= World Health Organization, ATPIII=, IDF= International Diabetes Federation
