# Supplementary material for: Associations of TERC Single Nucleotide Polymorphisms with Human Leukocyte Telomere Length and the Risk of Type 2 Diabetes Mellitus
Source: PLoS One. 2015 Dec 31;10(12):e0145721. doi: 10.1371/journal.pone.0145721 (PMC4705103; doi:10.1371/journal.pone.0145721)
Supplement: S3 Table — (DOCX) [file pone.0145721.s004.docx]

**S4 Table: The distribution of genotypes within the study population.**

| **Genotype** | **Controls**  **(n=245)** | **T2DM Patients (n=225)** | **MAF** | **HW**  ***p* value** | ***p* value trend^a^** |
| --- | --- | --- | --- | --- | --- |
| **rs16847897** | | | | | |
| GG | 118(47.6%) | 80(35.7%) |  |  |  |
| GC | 99(39.9%) | 105(46.9%) | C (36.6%) | 0.19 | 0.02 |
| CC | 31(12.5%) | 37(16.4%) |  |  |  |
| **rs12696304** | | | | | |
| CC | 118(48.2%) | 82(36.4%) |  |  |  |
| GC | 99(40.4%) | 106(47.1%) | G (35%) | 0.32 | 0.01 |
| GG | 28(11.4%) | 37(16.4%) |  |  |  |

MAF= Minor Allele Frequency, HW= Hardy Weinberg Equilibrium.

a Trend was calculated using goodness of fit tests.
