# Supplementary material for: Associations of TERC Single Nucleotide Polymorphisms with Human Leukocyte Telomere Length and the Risk of Type 2 Diabetes Mellitus
Source: PLoS One. 2015 Dec 31;10(12):e0145721. doi: 10.1371/journal.pone.0145721 (PMC4705103; doi:10.1371/journal.pone.0145721)
Supplement: S4 Table — (DOCX) [file pone.0145721.s005.docx]

**S5 Table: Frequency of metabolic syndrome in the study population according to *TERC***

**genotypes.**

|  | **rs16847897** | | | **rs12696304** | | |
| --- | --- | --- | --- | --- | --- | --- |
|  | GG | GC | CC | GG | GC | CC |
| **WHO** | | | | | | |
| **Metabolic Syndrome Positive** | 41 | 140 | 126 | 127 | 141 | 39 |
| **Metabolic Syndrome Negative** | 27 | 64 | 72 | 73 | 64 | 26 |
| **ATPIII** | | | | | | |
| **Metabolic Syndrome Positive** | 48 | 131 | 141 | 145 | 143 | 42 |
| **Metabolic Syndrome Negative** | 20 | 73 | 57 | 55 | 62 | 23 |
| **IDF** | | | | | | |
| **Metabolic Syndrome Positive** | 52 | 136 | 140 | 146 | 59 | 16 |
| **Metabolic Syndrome Negative** | 16 | 68 | 58 | 54 | 146 | 49 |

WHO= World Health Organization, ATPIII=, IDF= International Diabetes Federation
