# Supplementary material for: Associations of TERC Single Nucleotide Polymorphisms with Human Leukocyte Telomere Length and the Risk of Type 2 Diabetes Mellitus
Source: PLoS One. 2015 Dec 31;10(12):e0145721. doi: 10.1371/journal.pone.0145721 (PMC4705103; doi:10.1371/journal.pone.0145721)
Supplement: S5 Table — (DOCX) [file pone.0145721.s006.docx]

**S6 Table: Beta Coefficients [95CI] for associations of TERC SNPs rs16847897and rs12696304 with LTL, hTERT, anthropometric indices and metabolic factors of obesity.**

|  | LTL | hTERT | BMI | WC | AdipoQ | HMW-  AdipoQ | Insulin | | HOMA-IR |
| --- | --- | --- | --- | --- | --- | --- | --- | --- | --- |
|  | **β (95%CI)** | **β(95%CI)** | **β(95%CI)** | **β(95%CI)** | **β(95%CI)** | **β(95%CI)** | **β(95%CI)** | | **β(95%CI)** |
|  | **rs16847897** | | | | | | | | |
| **GG+GC** | 0.2*(0.1 to 0.6) | 0.1(0.1 to 0.6) | -0.2*(-0.1 to -0.6) | -0.2*(-0.1 to -0.6) | 0.1(0.1 to 0.6) | 0.1(0.0 to 0.6) | | -0.1(0.0 to -0.4) | -0.1(0.0 to -0.4) |
| **CC** | -0.5**(-0.1 to -0.7) | -0.2*(-0.1 to -0.7) | 0.6**(0.1 to 0.7) | 6.5**(1.1 to 8.7) | -0.3*(-0.1 to -0.7) | -0.2*(-0.1 to -0.5) | | 0.3*(0.3 to 0.8) | 0.6**(0.1 to 0.8) |
|  | **rs12696304** | | | | | | | | |
| **CC+CG** | 0.1(0.0 to 0.7) | 0.1(0.1 to 0.6) | -0.2*(-0.1 to -0.6) | -0.2*(-0.1 to -0.6) | 0.1(0.1 to 0.6) | 0.1(0.0 to 0.6) | -0.1(0.0 to -0.4) | | -0.1(0.0 to -0.4) |
| **GG** | -0.4**(-0.2 to -0.6) | -0.2*(-0.1 to -0.7) | 0.3*(0.1 to 0.7) | 1.4**(1.2 to 5.7) | -0.3*(-0.1 to -0.7) | -0.2(-0.1 to -0.5) | 0.4*(0.3 to 0.8) | | 0.4**(0.1 to 0.8) |

* *p*<0.05, ** *p*<0.01, *** *p*<0.001, **** *p*<0.0001, No * = No Significance.

β = Beta Coefficient.

LTL= Leukocyte Telomere Length, hTERT=Human Telomerase Reverse Transcriptase, BMI= Body Mass Index, WC =Waist Circumference, AdipoQ= Adiponectin, HMW-AdipoQ= High Molecular Weight Adiponectin, HOMA-IR= Homeostasis Model Assessment Insulin Resistance.
