# Supplementary material for: Associations of TERC Single Nucleotide Polymorphisms with Human Leukocyte Telomere Length and the Risk of Type 2 Diabetes Mellitus
Source: PLoS One. 2015 Dec 31;10(12):e0145721. doi: 10.1371/journal.pone.0145721 (PMC4705103; doi:10.1371/journal.pone.0145721)
Supplement: S6 Table — (DOCX) [file pone.0145721.s007.docx]

**S7 Table: Anthropometric and biochemical characteristics according to haplotypes.**

|  | CCGG | CCCC | GGGG |  |
| --- | --- | --- | --- | --- |
| Parameter | Mean±SD | Mean±SD | Mean±SD | *p*-value |
| Age (years) | 55.0±0.4 | 53.5±0.3 | 54.68 | NS |
| BMI (Kg/m2) | 30.8±8.2 | 26.9±8.4 | 28.7±5.3 | 0.03 |
| WC (cm) | 108.6±13.0 | 102.8±7.3 | 92.6±13.2 | 0.004 |
| HbA1c % | 8.2±0.5 | 7.9±0.7 | 7.5±0.5 | <0.0001 |
| FPG (mmol/L) | 8.5±3.5 | 5.8±0.6 | 5.0±0.6 | <0.0001 |
| LTL | 0.8±.01 | 1.03±0.1 | 1.5±0.1 | 0.02 |
| hTERT (ng/mL) | 21.8±5.5 | 23.7±6.9 | 28.1±5.4 | 0.005 |
| Total AdipoQ (ng/mL) | 6.1±1.1 | 7.0±1.3 | 6.7±0.8 | 0.009 |
| Insulin (µU/mL) | 12.8±2.7 | 10.1±2.8 | 10.6±1.8 | 0.03 |
| HOMA-IR | 5.0±2.2 | 2.6±0.8 | 2.4±0.5 | <0.0001 |

BMI= Body Mass Index, WC =Waist Circumference, HbA1c= Glycated Hemoglobin A1c,

FPG=Fasting Plasma Glucose, LTL= Leukocyte Telomere Length, hTERT=Human

Telomerase Reverse Transcriptase, AdipoQ= Adiponectin, HOMA-IR= Homeostasis Model

Assessment Insulin Resistance.
