# Supplementary material for: Associations of TERC Single Nucleotide Polymorphisms with Human Leukocyte Telomere Length and the Risk of Type 2 Diabetes Mellitus
Source: PLoS One. 2015 Dec 31;10(12):e0145721. doi: 10.1371/journal.pone.0145721 (PMC4705103; doi:10.1371/journal.pone.0145721)
Supplement: S1 Text — (DOC) [file pone.0145721.s008.doc]

**S1**

**Detailed protocol for Leukocyte Telomere Length (LTL) measurement:** For each sample, two qPCRs were performed on separate plates, with the same sample well position: the first one to amplify the telomeric DNA [T] and the second one to amplify a single-copy control gene [S] (36B4, acidic ribosomal phosphoprotein PO, located on chromosome 12) to normalize the starting amount of DNA. Both primers (Telomeric [tel1b: 5\-CGG TTT GTT TGG GTT TGG GTT TGG GTT TGG GTT TGG GTT-3\, tel2b: 5\-TCCCGACTATCCCTATCCCTATCCCTATCCCTATCCCTA-3\] and 36B4 [36b4μ: 5\- CAG CAA GTG GGA AGG TGT AAT CC-3\, 36b4d: 5\-CCC ATT CTA TCA TCA ACG GGT ACA A-3\]) were ordered from Integrated SIGMA technologies (Sigma – Aldrich, St. Louis, MO, USA) and were reconstituted at a final concentration of 100 μM. Each 96 well plate contained a seven-point standard curve (2-fold serial dilutions from 0.3125 to 20 ng/μl of DNA), which was prepared from a reference DNA sample (120 ng/μl) [pooled human DNA obtained from apparently healthy donors aged from 20 – 70 years] by serial dilutions (1:2) with MPW to allow the transformation of Cycle threshold (Ct) into nanograms of DNA. The final concentration of genomic DNA for all unknown samples in the experiment was adjusted to yield a uniform DNA concentration (20 ng/μl) before carrying on the experiment. The PCR thermal conditions for both telomeric and 36B4 PCR runs consisted of initial denaturation for 10 minutes at 95^0^C, followed by a total 40 cycles at 95^0^C for 15 seconds, and 60^0^C for 1 minute followed by melting (dissociation) curves to avoid false positive signals caused by the formation of primer-dimer products and hence to ensure specificity. T/S ratio was calculated as the amount of telomeric DNA (T) divided by the amount of single copy control gene DNA (S). Once this was established, the reproducibility of the assay was tested. For that purpose, 20 randomly chosen DNA samples were run in duplicates on two consecutive days. There was significant linearity between the mean telomere length measurements obtained on the two different days in linear regression analysis (R^2^=0.97, p<0.0001). The intra- and inter-assay variability (CV) for singleplex qPCR was 2.1% and 3%, respectively. Duplicate values between runs which varied by more than 5% above or below the calculated CV, were assayed a third time and the two closest values were used.

**Detailed protocol for genotyping SNPs near *TERC* using Allelic Discrimination:** Genomic DNA for the AD experiment was isolated from blood as was described in section. DNA samples used in AD experiment were insured to have an A260/280 ratio greater than 1.7 and are intact as visualized by gel electrophoresis. Aafter testing different amounts of template (10ng, 20ng, 30ng, 40ng and 50 ng), it was shown that the 50ng of template is the optimal amount. Hence, the final concentration of genomic DNA for all samples in the experiment was (50 ng/μl). The 96-well reaction plate for the AD assay was designed to contain No template Controls (NTCs) and unknown genomic DNA samples. Firstly the number of reactions to be performed for each assay was calculated and the volume of components needed for all wells on the reaction plate was calculated as well. Extra reactions were added to provide excess volume for the loss that occurs during reagent transfers. The bottle of 2X TaqMan Genotyping Assay Master Mix, No AmpErase UNG was mixed gently to re-suspend. Then, 40X SNP genotyping Assay Mix was mixed and centrifuged briefly. The volumes required for all wells on the reaction plate (plus additional reactions to compensate for reagent transfer loss) of 2X TaqMan Genotyping Assay Master Mix, No AmpErase UNG, 40X SNP genotyping Assay Mix and DNase Free Water into sterile micro-centrifuge tube which was then capped. The prepared reaction mix tube was inverted and centrifuged briefly to spin down the contents and to eliminate air bubbles. 15 μl of reaction mix was transferred into each well in a 96-well reaction plate. After thawing frozen pre-diluted DNA samples, the samples were re-suspended by vortexing and then centrifuging the tubes briefly. Then, 5 μl of pre-diluted (10ng/μl) genomic DNA was added to the corresponding wells on the reaction plate. Upon the addition of all DNA samples, the wells were inspected for uniformity of volume. The reaction plate was covered with an optical adhesive cover and was kept on ice until loading in the 7500 fast system. The plate was then vortexed to mix the wells and then centrifuged briefly to spin down the contents and to eliminate any air bubbles. The reaction plate was loaded into the thermal cycler, and the run was started. Using 7500 Fast system software, a pre-Read run was initiated once the markers and detectors were programmed. This was followed by performing an amplification run. After an AD post-read run was successfully completed, the 7500 Fast SDS software analyzed raw data which were expressed in terms of fluorescence versus filters. During the analysis, the raw data were converted by SDS software to pure dye components using the extracted pure dye standards. After identifying the dye components, the SDS software determined the contribution of each dye in the raw data using the multi-component algorithm. Amplifications curves for each sample were further examined to verify results for allele calling.
